# Early‐life internalizing behavioral trajectories and childhood‐onset asthma: An epigenetic link

**DOI:** 10.1111/pai.70436

**Published:** 2026-08-16

**Authors:** Jisun Yoon, Seung‐Hwa Lee, Jiseon Kim, Kyungmo Hong, So‐Yeon Lee, Hea Young Oh, Sungsu Jung, Da Kyeong Lee, Eom Ji Choi, Ji Soo Park, Youn Ho Shin, Kyung Won Kim, Kangmo Ahn, Jihyun Kim, Yee‐Jin Shin, Kyung‐Sook Lee, Dong In Suh, Soo‐Jong Hong, the Cohort for Childhood Origin of Asthma and Allergic Diseases (COCOA) and Panel Study on Korean Children (PSKC) Study Groups, Jisun Yoon, Seung‐Hwa Lee, Jiseon Kim, Kyungmo Hong, So‐Yeon Lee, Hea Young Oh, Sungsu Jung, Da Kyeong Lee, Eom Ji Choi, Ji Soo Park, Youn Ho Shin, Kyung Won Kim, Kangmo Ahn, Jihyun Kim, Yee‐Jin Shin, Kyung‐Sook Lee, Dong In Suh, Soo‐Jong Hong

**Affiliations:** ^1^ Department of Pediatrics Asan Medical Center, University of Ulsan College of Medicine Seoul Republic of Korea; ^2^ Asan Institute for Life Sciences Asan Medical Center Seoul Republic of Korea; ^3^ Major in Psychotherapy, Graduate School of Health & Welfare Baekseok University Cheonan Republic of Korea; ^4^ Institute for Innovation in Digital Healthcare Yonsei University Seoul Republic of Korea; ^5^ Department of Pediatrics Pusan National University Yangsan Hospital Yangsan Republic of Korea; ^6^ Department of Pediatrics Gangnam CHA Medical Center, CHA University School of Medicine Seoul Republic of Korea; ^7^ Department of Pediatrics Seoul National University College of Medicine Seoul Republic of Korea; ^8^ Department of Pediatrics The Catholic University of Korea Seoul Republic of Korea; ^9^ Department of Pediatrics Yonsei University College of Medicine Seoul Republic of Korea; ^10^ Department of Pediatrics Samsung Medical Center, Sungkyunkwan University School of Medicine Seoul Republic of Korea; ^11^ Department of Psychiatry Yonsei University College of Medicine Seoul Republic of Korea; ^12^ Department of Rehabilitation Hanshin University Osan Republic of Korea; ^13^ Department of Pediatrics, Humidifier Disinfectant Health Center National Medical Center Seoul Republic of Korea

**Keywords:** anxiety, asthma, birth cohort, DNA methylation, internalizing behavior, mental health, preschool child

## Abstract

**Background:**

Behavioral problems and asthma in childhood are considered related despite the debatable causal relationship and unknown mechanisms. We investigated whether early‐life behavioral problem trajectories affect the risk of childhood asthma and ascertained potential DNA‐methylation mechanisms.

**Methods:**

Based on two independent birth cohorts, this study included 1041 and 1647 children aged 6–10 and 6–7 years in the Cohort for Childhood Origin of Asthma and Allergic Diseases (COCOA) study and Panel Study on Korean Children (PSKC), wherein asthma was defined by a physician's diagnosis and parental questionnaire, respectively. The Korean version of the Child Behavior Checklist (CBCL) was administered at ages 2–6 years. The CBCL trajectories were identified using a latent generalized mixture model. Blood samples from 7‐year‐old participants were used for DNA‐methylation profiling and quantifying metabolites and proteins.

**Results:**

Trajectories with high internalizing behavioral problem scores in preschool age were significantly associated with childhood‐onset asthma. Increased CBCL anxiety/depression scores at age 2 (COCOA) and somatization scores at ages 4 and 6 (COCOA and PSKC) significantly increased the risk of asthma symptoms and current asthma. In an exploratory sub‐study, DNA‐methylation analysis suggested hypomethylation of *HAL* and *MAD1L1* in children with co‐occurring asthma and behavioral problems, accompanied by higher HAL protein and lower circulating histidine.

**Conclusion:**

Early‐life internalizing behavioral problem trajectories precede childhood asthma, which may be mediated by methylation, especially of *HAL* and *MAD1L1*.

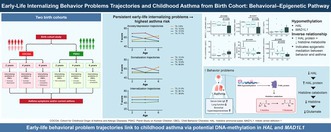

## Graphical abstract

Persistently elevated early‐life internalizing behavioral problems precede childhood‐onset asthma, with *HAL* and *MAD1L1* hypomethylation as a candidate shared mechanism. CBCL, Child Behavior Checklist; COCOA, Cohort for Childhood Origin of Asthma and Allergic Diseases; *HAL*, histidine ammonia‐lyase; *MAD1L1*, mitotic arrest deficient 1‐like 1; PSKC, Panel Study of Korean Children.

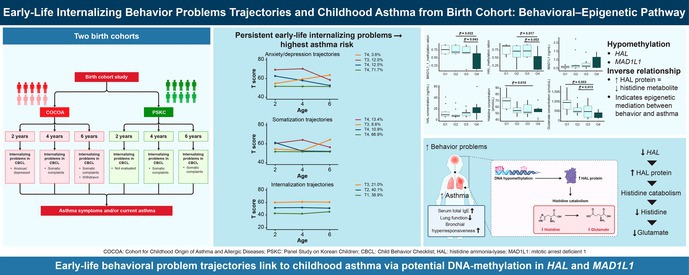

**Abbreviations**
CBCL: Child Behavior Checklist
COCOA: The Cohort for Childhood Origin of Asthma and allergic diseases
ELISA: Enzyme‐linked immunosorbent assay
HAL: histidine ammonia‐lyase
MAD1L1: mitotic arrest deficient 1 like 1
PSKC: The Panel Study of Korean Children

**Key message**

Early‐life behavioral problem trajectories are linked to childhood asthma via potential DNA‐methylation in *HAL* and *MAD1L1*. Continuous early‐life mental health support may be important to prevent the development of childhood asthma.

## 1 INTRODUCTION

Asthma is a heterogeneous chronic airway disease characterized by recurrent, reversible bronchial obstruction.^1^ Given its increasing prevalence, it constitutes a significant public health burden in the pediatric population worldwide.^2^ Childhood‐onset asthma affects the mental health problems of the affected children and their families.^3^ Mental health problems, including depression and anxiety, have become highly prevalent in childhood over the past few years.^4^ Thus, research on the interactions between allergy‐related inflammatory or immunological factors and psychiatric disorders, such as depression and anxiety, has been increasing in recent years.^5^, ^6^ The interrelationship between these two fields, childhood asthma and mental health problems, which confer a global social burden, especially on the younger generation, warrants attention.^7^ Although the coexistence of allergic diseases and mental health disorders has been frequently reported, and their prevalence continues to rise, the underlying evidence supporting this association remains limited.^8^ Therefore, the preceding relationship and shared mechanisms underlying the association between allergic diseases and psychiatric disorders need to be clarified.

Despite accumulating evidence of the association of children's behavioral problems with allergic diseases,^9^ the causality or preceding relationship remains unclear, and whether confounding factors or reverse causality could explain the observed association has not been fully demonstrated. Only a limited number of studies have provided causal evidence that childhood mental health problems may precede and contribute to the development of allergic diseases, particularly asthma. A longitudinal population‐based birth‐cohort study showed that children's mental health problems precede the development of preschool childhood wheezing.^10^ However, the study's limitations included a single assessment of children's behavioral problems at age 3 and the evaluation of wheezing episodes at ages 3 and 5 with relatively short intervals. Moreover, not all wheezing is attributable to pediatric asthma.^11^ Recent large‐scale prospective studies from two German birth cohorts (GINIplus and LISA) also attempted to demonstrate that psychopathological symptoms at age 10 were associated with asthma at age 15, particularly for non‐atopic asthma, showing significant interaction effects with the onset of puberty.^12^ However, these studies were limited by the lack of plausible mechanistic explanations supporting the causal relationship. Notably, previous cohort studies did not explicitly evaluate early‐life stress exposures nor apply longitudinal trajectory modeling of behavioral symptoms, further limiting causal inference about timing and mechanisms of asthma development.

The exact mechanisms underlying the above‐described associations are incompletely understood. Despite the positing of putative mechanisms, such as the sharing of allergic inflammation in chronic inflammation mediated by inflammatory cytokines and other immune factors that modulate neurotransmitter systems and brain function,^9^ no study has provided longitudinal clinical findings and a comprehensive elucidation of the underlying shared mechanisms. As environmental factors induce systemic inflammation and epigenetic changes,^13^ we posited that early‐life behavioral problems may, via epigenetic changes, contribute to the etiology of childhood asthma.

By replicating two independent general population‐based birth‐cohort studies, which comprised longitudinal trajectories of the Child Behavior Checklist (CBCL) scores, we aimed to investigate early‐life behavioral problem trajectories and subsequent childhood asthma. To overcome the limitations of previous studies, we ascertained the potentially shared DNA‐methylation mechanisms.

## 2 METHODS

### 2.1 Participants

The Cohort for Childhood Origin of Asthma and Allergic Diseases (COCOA) Study is a general‐population‐based prospective birth‐cohort study comprising 2471 newborns to identify early‐life risk factors for childhood allergic diseases.^14^ The Panel Study of Korean Children (PSKC), another independent birth‐cohort study, surveyed longitudinal child development, childcare behaviors, and education in 2078 mother–child dyads recruited using 2‐step stratified random sampling from 30 obstetric hospitals nationwide.^15^ Table S1 summarizes the demographic characteristics of the COCOA and PSKC cohorts, and the Institutional Review Board of Asan Medical Center approved each study protocol (2008‐0616 and 2015‐0907, respectively). The studies were conducted in accordance with the Declaration of Helsinki, and the participants (and parents and guardians, as relevant) provided written informed consent to participate.

### 2.2 Study design and analytic framework

COCOA served as the primary discovery cohort (three CBCL waves at 2, 4, and 6 years; physician‐diagnosed asthma with objective testing; stored biospecimens), and PSKC as an independent replication cohort (a larger, separately recruited sample with CBCL at 4 and 7 years). Two complementary analyses were applied: group‐based trajectory modeling to characterize longitudinal patterns, and time‐point‐specific logistic regression to localize age windows and test replication. Trajectory modeling, which requires at least three repeated measures, was applied to COCOA only. The overall study procedures and conduct of the study are summarized in Figure S1.

### 2.3 Evaluation of childhood asthma

In COCOA, asthma was ascertained at 6–10 years by physician diagnosis, with symptoms captured by the modified ISAAC questionnaire; “asthma symptoms” denoted wheezing in the previous 12 months and “current asthma” a lifetime physician diagnosis plus wheezing in the previous 12 months.^16^ Spirometry, methacholine challenge, serum IgE, and eosinophil levels were evaluated at 7 years among COCOA participants as clinical parameters.^17^ In PSKC, asthma was ascertained at 6–7 years by parent‐reported physician diagnosis, with identical symptom definitions.

### 2.4 Assessment of children's internalizing problems

The CBCL (described in Supplementary Methods: Appendix S1) has wide application for evaluating mental health and behavioral difficulties among children and provides standardized T‐scores that enable inter‐age‐group comparisons.^18^ Children with borderline‐to‐clinical range (≥65) scores in the internalization subcategories (emotionally reactive, anxious/depressed, somatic complaints, and withdrawn/depressed) were diagnosed with behavioral problems.

Longitudinal trajectories of T‐scores from internalization subcategories (internalization, anxiety/depression, and somatic symptoms) were identified using group‐based trajectory modeling. T‐scores at 2, 4, and 6 years of age were used as repeated measures at each time point. The appropriate order and number of trajectories were determined using the significant term and lowest absolute Bayesian information criterion score, respectively.^19^

### 2.5 DNA‐methylation experiments

In the COCOA Study, 39 participants underwent blood sampling at the age of 7 years, and DNA was extracted using the QIAamp DNA Blood Mini Kit (Qiagen, Hilden, Germany) for DNA‐methylation profiling using the Infinium MethylationEPIC BeadChip kits (Illumina, San Diego, CA, USA).^20^

In this exploratory sub‐study, among COCOA participants who provided a blood sample at 7 years with complete CBCL and asthma data, four pre‐specified, group‐balanced groups were assembled by design (low‐CBCL/non‐asthma, *n* = 10; low‐CBCL/asthma, *n* = 10; high‐CBCL/non‐asthma, *n* = 10; high‐CBCL/asthma, *n* = 9), balanced on sex, gestational age, maternal age, birth weight, and maternal depression (all *p* ≥ .20; Table S2). This design was intended for biomarker discovery and contrast, not for population‐level estimation. Participants with borderline/clinical and normal T‐scores were assigned to the high CBCL and low CBCL groups, respectively. The procedures are described in the Supplementary Methods: Appendix S1.

### 2.6 Targeted metabolite quantification and ELISA‐based protein measurement

Plasma levels of histidine and glutamate, two amino acids in the histidine metabolic pathway, were quantified by liquid chromatography–tandem mass spectrometry (LC–MS/MS) using multiple‐reaction monitoring, as previously described.^20^ Serum protein concentrations of histidine ammonia‐lyase (HAL; abx156887, Abbexa) and mitotic arrest deficient 1‐like 1 (MAD1L1; abx385115, Abbexa) were determined by commercial enzyme‐linked immunosorbent assay (ELISA) kits according to the manufacturer's instructions (Supplementary Methods: Appendix S1). Histamine was not directly measured in this study.

### 2.7 Statistical analysis

**FIGURE 1:**
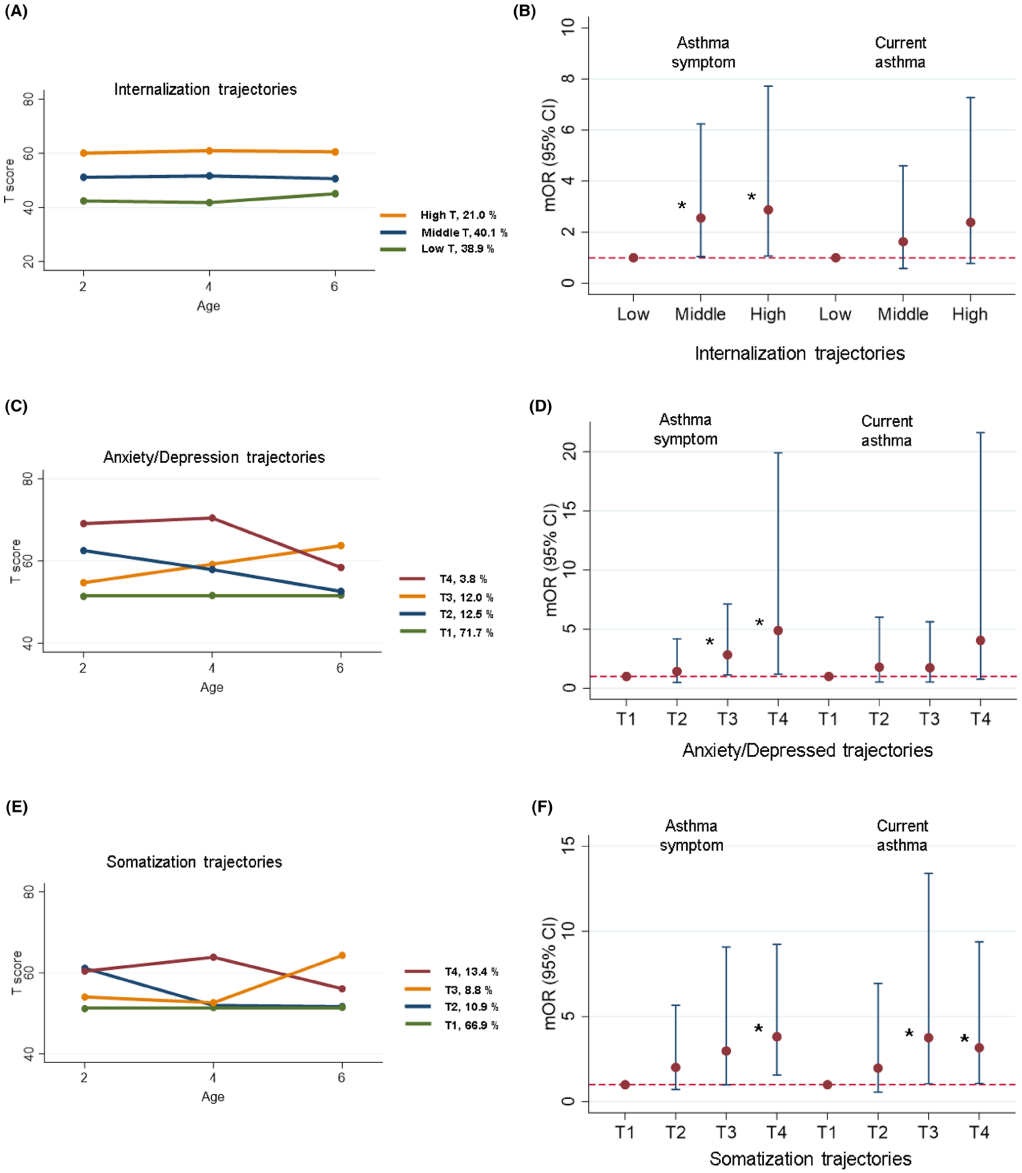
Trajectories of internalizing behavior problems and risk factors for asthma in the COCOA Study. The analysis was adjusted for sex, birth weight, gestational age, maternal age, BMI, breastfeeding for at least 6 months, parental history of allergic diseases, maternal education levels, and any wheezing episodes during ages 6–24 months.

An independent sample *t*‐test was used to ascertain intergroup differences in the CBCL scores of children with and without asthma. Odds ratios (OR) and 95% confidence intervals (CI) of asthma risk were calculated using logistic regression models adjusted (adjusted odds ratio 1, aOR1) for confounders, such as sex, birth weight, gestational age, maternal age, body mass index (BMI), breastfeeding for at least 6 months, parental history of allergic diseases, maternal education levels, and maternal CES‐D score (caregiver‐administered test during the preschool period). We additionally considered any wheezing episodes before 2 years of age as a confounding factor (adjusted odds ratio 2, aOR2) to distinguish bronchiolitis from asthma in early infancy. Statistical significance was set at *p* < .05, and analyses were performed using SPSS version 22 (SPSS, Chicago, IL, USA), SAS version 9.3 (SAS Institute, Cary, NC, USA), and GraphPad Prism 5.0 (GraphPad, San Diego, CA, USA) using one‐way analysis of variance (Tukey's multiple‐comparison test). Trajectory modeling was performed using Stata 16.0 software (Stata Corp., College Station, TX, USA). The time‐point–specific logistic‐regression models (CBCL T‐scores at each age; Tables 1 and 2) and the trajectory‐group models (T1–T4 as the exposure; Figure 1) were fitted separately. For the group‐based trajectory models, internalizing T‐scores at 2, 4, and 6 years were entered as repeated measures, and the polynomial order and the number of groups were selected by the Bayesian information criterion together with the significance of the highest‐order term, with model adequacy confirmed by an average posterior probability of group membership ≥0.7. In the exploratory methylation screen, differentially methylated CpG sites were defined by *p* < .05 and a mean methylation difference |Δβ| >0.2 (Supplementary Methods: Appendix S1), and associations among methylation, protein, metabolite, and clinical or CBCL variables were assessed using Spearman's rank correlation. Candidate signals were instead required to show biological concordance across the methylation, protein, and metabolite layers.

**TABLE 1:** Association of internalizing behavior problems at 2, 4, and 6 years with childhood‐onset asthma in the COCOA Study.

CBCL 1.5–5	T‐score	Two years				Four years				Six years			
CBCL 6–18		Asthma symptoms		Current asthma		Asthma symptoms		Current asthma		Asthma symptoms		Current asthma	
Internalizing Problem areas		aOR^a^ (95% CI)	aOR^b^ (95% CI)	aOR^a^ (95% CI)	aOR^b^ (95% CI)	aOR^a^ (95% CI)	aOR^b^ (95% CI)	aOR^a^ (95% CI)	aOR^b^ (95% CI)	aOR^a^ (95% CI)	aOR^b^ (95% CI)	aOR^a^ (95% CI)	aOR^b^ (95% CI)
Internalizing	<60	Ref.	Ref.	Ref.	Ref.	Ref.	Ref.	Ref.	Ref.	Ref.	Ref.	Ref.	Ref.
	≥60	1.11 (0.56–2.19)	1.09 (0.55–2.16)	1.24 (0.51–2.98)	1.24 (0.51–3.03)	1.37 (0.64–2.93)	1.14 (0.52–2.50)	1.03 (0.35–3.02)	0.90 (0.30–2.67)	1.52 (0.77–3.00)	1.52 (0.76–3.05)	1.00 (0.36–2.82)	1.00 (0.35–2.83)
Emotionally reactive	<65	Ref.	Ref.	Ref.	Ref.	Ref.	Ref.	Ref.	Ref.	Ref.	Ref.	Ref.	Ref.
	≥65	1.59 (0.63–4.01)	1.66 (0.65–4.23)	0.97 (0.22–4.32)	1.05 (0.23–4.74)	0.52 (0.12–2.28)	0.40 (0.09–1.83)	—	—	—	—	—	—
Anxious/depressed	<65	Ref.	Ref.	Ref.	Ref.	Ref.	Ref.	Ref.	Ref.	Ref.	Ref.	Ref.	Ref.
	≥65	2.44 (1.05–5.67)	2.59 (1.10–6.10)	3.75 (1.39–10.08)	4.23 (1.55–11.53)	1.30 (0.41–4.08)	1.06 (0.32–3.50)	1.47 (0.37–5.78)	1.16 (0.28–4.90)	1.35 (0.60–3.04)	1.29 (0.56–2.97)	1.23 (0.40–3.77)	1.17 (0.38–3.65)
Somatic complaints	<65	Ref.	Ref.	Ref.	Ref.	Ref.	Ref.	Ref.	Ref.	Ref.	Ref.	Ref.	Ref.
	≥65	0.73 (0.17–3.21)	0.74 (0.17–4.99)	0.66 (0.08–5.18)	0.73 (0.09–5.69)	3.62 (1.34–9.81)	3.76 (1.34–10.54)	4.95 (1.45–16.94)	5.17 (1.46–18.33)	4.36 (1.66–11.42)	5.91 (2.17–16.10)	3.00 (0.80–11.33)	3.86 (1.01–15.02)
Withdrawn/depressed	<65	Ref.	Ref.	Ref.	Ref.	Ref.	Ref.	Ref.	Ref.	Ref.	Ref.	Ref.	Ref.
	≥65	1.40 (0.60–3.26)	1.40 (0.59–3.32)	1.44 (0.48–4.29)	1.43 (0.47–4.34)	0.73 (0.16–3.28)	0.65 (0.14–3.02)	1.18 (0.23–6.00)	1.18 (0.23–6.08)	2.28 (0.91–5.67)	2.32 (0.90–5.96)	4.12 (1.42–11.96)	4.20 (1.41–12.52)

Abbreviation: COCOA, Cohort for Childhood Origin of Asthma and allergic diseases.

^a^ Adjusted by sex, birth weight, gestational age, maternal age and BMI, breast milk feeding at least 6 months, parental history of allergic diseases, maternal education levels, maternal CES‐D score (at children's age 2, 4, or 6).

^b^ Additionally adjusted by any wheezing episodes during 6 months to 2 years of age.

**TABLE 2:** Association of internalizing behavioral problems at 4 and 7 years with childhood‐onset asthma in the PSKC Study.

CBCL 1.5–5	T‐score	Four years				Seven years			
CBCL 6–18		Asthma symptoms		Current Asthma		Asthma symptoms		Current Asthma	
Internalizing Problem areas		aOR^a^ (95% CI)	aOR^b^ (95% CI)	aOR^a^ (95% CI)	aOR^b^ (95% CI)	aOR^a^ (95% CI)	aOR^b^ (95% CI)	aOR^a^ (95% CI)	aOR^b^ (95% CI)
Internalizing	<60	Ref.	Ref.	Ref.	Ref.	Ref.	Ref.	Ref.	Ref.
	≥60	1.06 (0.57–1.97)	0.82 (0.43–1.59)	1.41 (0.60–3.32)	1.20 (0.50–2.87)	0.82 (0.43–1.59)	0.99 (0.50–1.97)	1.86 (0.82–4.23)	1.70 (0.72–3.98)
Emotionally reactive	<65	Ref.	Ref.	Ref.	Ref.	Ref.	Ref.	Ref.	Ref.
	≥65	0.44 (0.11–1.87)	0.38 (0.09–1.62)	0.57 (0.08–4.41)	0.51 (0.07–3.93)	—	—	—	—
Anxious/depressed	<65	Ref.	Ref.	Ref.	Ref.	Ref.	Ref.	Ref.	Ref.
	≥65	0.47 (0.11–1.97)	0.41 (0.10–1.75)	0.59 (0.08–4.41)	0.54 (0.07–4.09)	1.23 (0.53–2.85)	1.23 (0.53–2.85)	1.17 (0.35–3.98)	1.11 (0.32–3.88)
Somatic complaints	<65	Ref.	Ref.	Ref.	Ref.	Ref.	Ref.	Ref.	Ref.
	≥65	2.15 (1.02–4.54)	1.79 (0.82–3.90)	2.67 (0.98–7.23)	2.15 (0.75–5.94)	2.89 (1.22–6.82)	2.98 (1.21–7.32)	4.35 (1.54–12.31)	4.38 (1.45–13.17)
Withdrawn/depressed	<65	Ref.	Ref.	Ref.	Ref.	Ref.	Ref.	Ref.	Ref.
	≥65	1.17 (0.52–2.62)	1.22 (0.53–2.79)	1.57 (0.54–4.59)	1.66 (0.55–4.96)	1.35 (0.50–3.62)	1.35 (0.50–3.62)	2.65 (0.89–7.90)	2.50 (0.79–7.92)

Abbreviation: PSKC, Panel Study on Korean Children.

^a^ Adjusted by sex, birth weight, gestational age, maternal age and BMI, breast milk feeding at least 6 months, parental history of allergic diseases, maternal education levels, maternal prenatal Kessler score.

^b^ Additionally adjusted by any wheezing episodes during 6 months to 2 years of age.

## 3 RESULTS

### 3.1 Longitudinal trajectories of internalizing behavioral problems and the risk of asthma

Longitudinal trajectory analysis of COCOA participants showed that the internalization T‐scores could be classified into three groups (the low, middle, and high trajectory groups of T1, T2, and T3, respectively), whereas the internalizing subcategories could be longitudinally classified into four groups, from the lowest (T1) to the highest (T4) (Figure 1). Children with consistently highest scores of internalizing through ages 2–6 years (T3) had a significantly increased risk of asthma symptoms (aOR 2.88, 95% CI 1.07–7.72). Compared to T1, T2 had an increased risk of asthma symptoms, albeit with slightly lower ORs than T3 (aOR 2.56, 95% CI 1.05–6.24). A similar trend was observed in the current asthma group, where the group with higher T‐scores exhibited gradually increasing ORs compared to the group with the lowest T‐scores, although the difference was not significant (Figure 1A,B).

Analyses of the trajectories for anxiety/depression at ages 2, 4, and 6 revealed four distinct groups. Compared to the lowest T‐score group, T1, the ORs for asthma symptoms gradually increased in other groups. Particularly, the aOR for group T3, characterized by increasing T‐scores with higher age, was 2.83 (95% CI 1.13–7.12). T4, which consistently had high scores at ages 2 and 4, showed the most significant increase in the risk for future asthma symptoms (aOR 4.88, 95% CI 1.19–19.90; Figure 1C,D). Notably, the higher risk observed in T4 compared with T3 suggests that persistently elevated internalizing symptoms in early life may confer a greater risk of subsequent asthma than symptoms that increase later in childhood.

Lastly, in the somatization category, T4, which consistently had higher T‐scores at ages 2 and 4 years, exhibited a significant increase in the rates of both asthma symptoms and current asthma (aOR [95% CI] 3.81 [1.57–9.23] and 3.17 [1.07–9.39], respectively). Thereafter, the group with initially lower T‐scores at ages 2 and 4 but the highest scores at age 6 (T3) showed a significant association with current asthma and asthma symptoms (aOR 3.75, 95% CI 1.05–13.40; Figure 1E,F). No group showed a significant association with asthma in the withdrawn/depressed category of behavioral problems (data not shown).

In summary, a dose‐dependent trend in asthma onset was observed as total internalizing problems, anxiety/depression, and somatization increased longitudinally, with the highest risk observed in children with persistently elevated symptoms from early life (T4), suggesting a greater vulnerability compared with those showing a later increase in symptoms (T3).

### 3.2 Association of internalizing behavior problems with childhood asthma

Children with asthma had higher CBCL scores for all internalizing‐problems categories than controls in COCOA and PSKC. Especially, somatic complaints, anxious/depressed, and withdrawn/depressed categories were significantly different from controls (Tables S3 and S4).

An increased anxious/depressed score in the CBCL at 2 years of age significantly increased the risks (aOR1 [95% CI]) of asthma symptoms and current asthma (2.44 [1.05–5.67] and 3.75 [1.39–10.08], respectively) that were maintained and strongly increased after adjustment for wheezing before 2 years of age (aOR2 [95% CI] 2.59 [1.10–6.10] and 4.23 [1.55–11.53], respectively). Higher scores of somatic complaints at age 4 and 6 years were associated with childhood‐onset asthma symptoms (aOR1 [95% CI] 3.62 [1.34–9.81] and 4.36 [1.66–11.42], respectively), and additionally adjusting for wheezing episodes during early infancy strengthened these risks (aOR2 [95% CI] 3.76 [1.34–10.54] and 5.91 [2.17–16.10], respectively), with similar trends in the current asthma group at 4 and 6 years (aOR2 [95% CI] 5.17 [1.46–18.33] and 3.86 [1.01–15.02], respectively). High scores of withdrawn/depressed states at 6 years of age increased the risk of current asthma after childhood (aOR2 4.20, 95% CI 1.41–12.52; Table 1).

Logistic regression in the PSKC showed a significant association between asthma symptoms and higher‐level somatic complaints at 4 years of age (aOR1 2.15, 95% CI 1.02–4.54), although this association disappeared upon adjusting for wheezing during infancy. However, somatic complaints at age 7 significantly increased the risk of asthma symptoms with both adjusting methods (aOR1 2.89, 95% CI 1.22–6.82; aOR2 2.98, 95% CI 1.21–7.32). Lastly, higher‐level somatic complaints at 7 years of age were strongly associated with current asthma in childhood (aOR1 4.35, 95% CI 1.54–12.31; aOR2 4.38, 95% CI 1.45–13.17; Table 2).

### 3.3 Differences in DNA methylation states at 7 years of age are stratified by early‐life behavioral problems and childhood asthma

We analyzed DNA methylation in COCOA participants to identify the mechanisms underlying mental health problems and childhood asthma. In total, 51 genes (25 hypomethylated and 26 hypermethylated) exhibited significantly different methylation patterns in participants with high CBCL scores and children with asthma (Table S5). These 51 aberrantly methylated genes were used to conduct functional annotation enrichment analysis^21^ to identify the molecular functions of risk genes associated with high CBCL scores and asthma. In the disease‐terms category,^22^ 11 aberrantly methylated genes (*PRR12*, *MAD1L1*, *CTNNA2*, *BRSK2*, *GYS2*, *LSM1*, *PRKAG2*, *NTM*, *DPYSL2*, *HAL*, and *C1QTNF7*) were mainly associated with abnormal behavior, mental disorders, and neuropsychiatric diseases (PSYCH; Figure S2).

**FIGURE 2:**
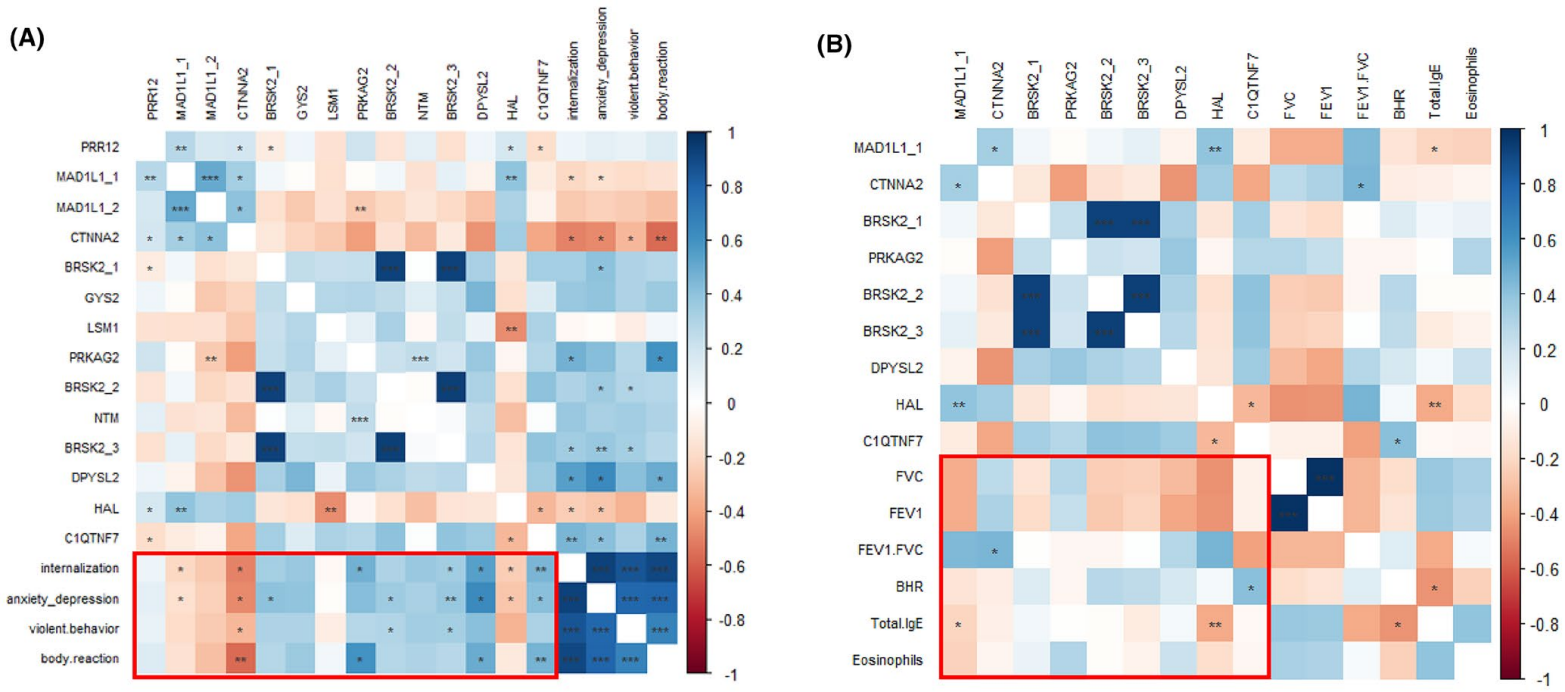
Correlation between 14 methylated genes and Child Behavior Checklist (CBCL) internalization categories, pulmonary functions, and allergic parameters. Seven genes (*MAD1L1*, *CTNNA2*, *BRSK2*, *PRKAG2*, *DPYSL2*, *HAL*, and *C1QTNF7*) showed significant correlations with CBCL scores at 6 years of age (A). *HAL* and *MAD1L1* genes showed significant correlations with participants' serum total IgE level at 7 years of age. *CTNNA2* and *C1QTNF7* showed significant correlations in FEV_1_/FVC and BHR (B). FVC (Forced vital capacity), FEV_1_ (Forced expiratory volume in the first second), BHR (Bronchial hyperresponsiveness).

We performed correlation analyses for clinical parameters, including CBCL categories and the methylation degrees of the 14 genes that were identified. Of these 14 genes, 7 (*MAD1L1*, *CTNNA2*, *BRSK2*, *PRKAG2*, *DPYSL2*, *HAL*, and *C1QTNF7*) showed significant correlations with CBCL scores at 6 years of age (Figure 2). *HAL* and *MAD1L1* genes showed significant correlations with the participant's serum total IgE level at 7 years of age. *CTNNA2* and *C1QTNF7* showed significant correlations in the ratio of the forced expiratory volume in the first second to the forced vital capacity of the lungs (FEV_1_/FVC) and bronchial hyperresponsiveness (BHR).

### 3.4 Differences in protein and metabolite levels caused by the hypomethylated *HAL* and *MAD1L1* genes in participants with high behavioral problems and asthma

**FIGURE 3:**
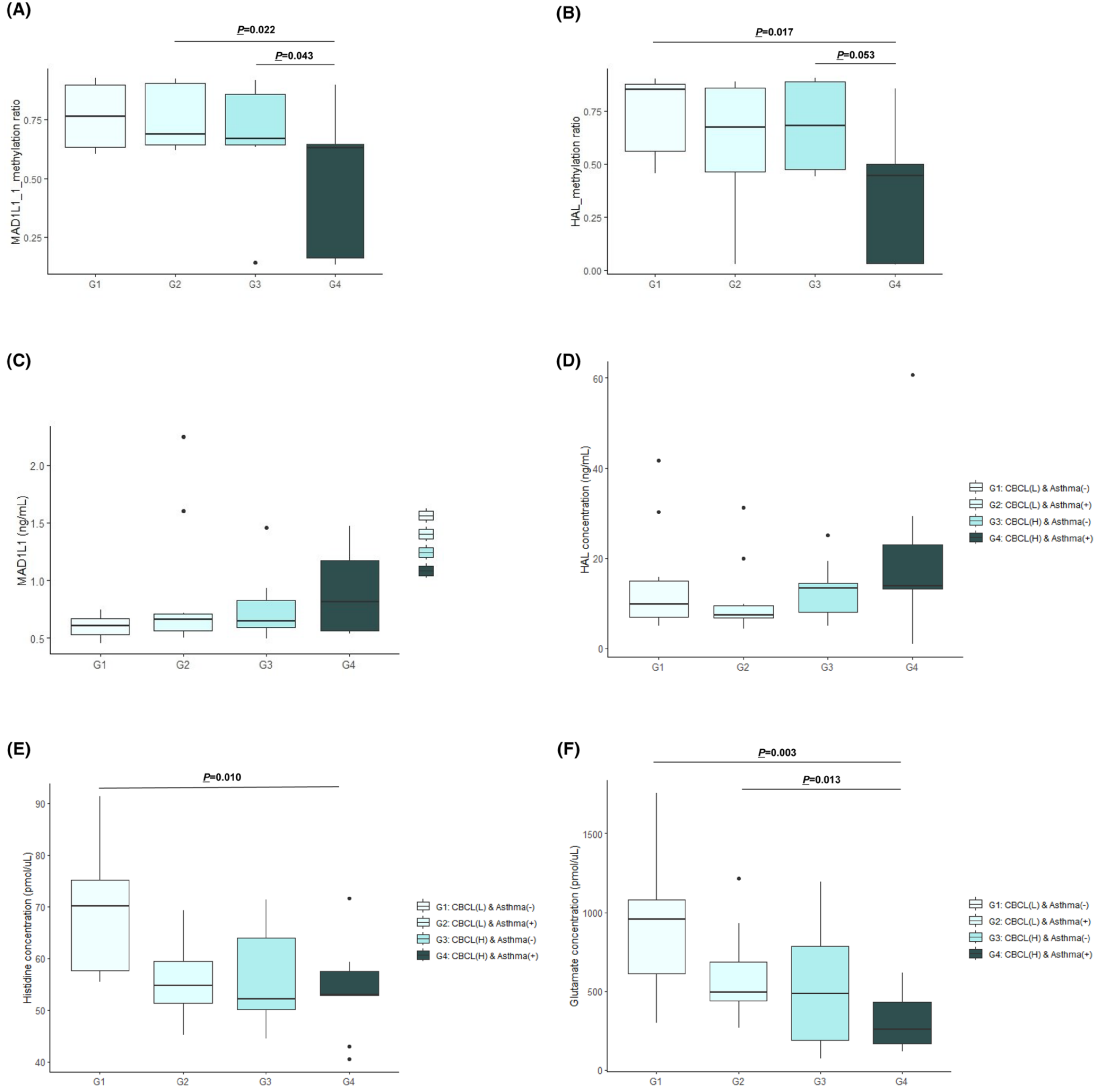
Methylation level (A and B) and protein concentration (C and D) of risk genes according to children's internalizing behavior problems and asthma, and the association with the concentrations of histidine metabolic pathway‐related metabolites (E and F).

**FIGURE 4:**
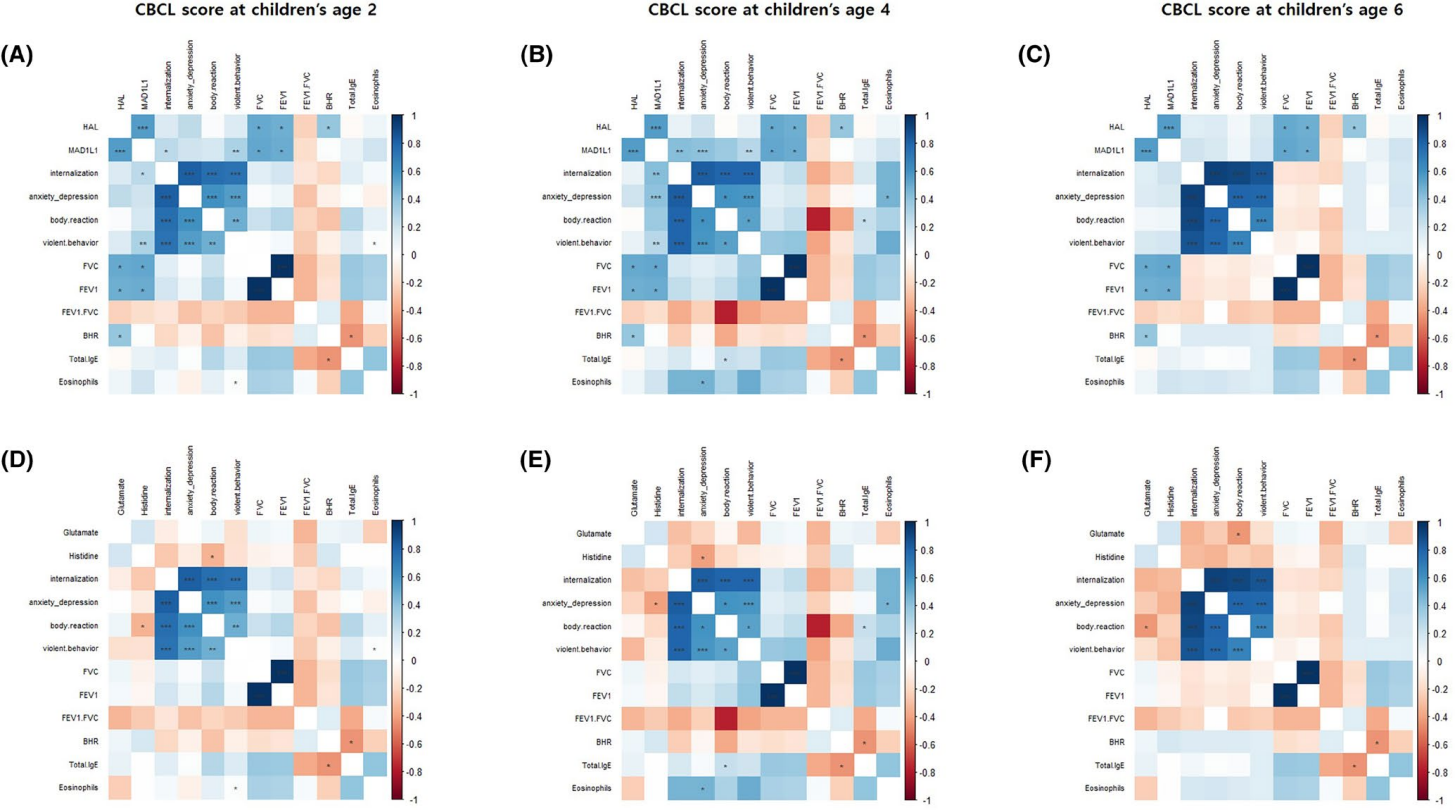
Correlations of HAL and MAD1L1 protein levels (A–C) and their corresponding metabolites, glutamate and histidine (D–F), with CBCL scores assessed at ages 2, 4, and 6 years and clinical parameters measured at age 7.

Compared to healthy controls, patients with high CBCL scores and asthma had significantly hypomethylated *HAL* and *MAD1L1* (Figure 3A,B); this pattern was consistently observed as higher HAL and MAD1L1 protein levels in children with asthma and severe behavioral problems (Figure 3C,D). Because *HAL* encodes histidase, the rate‐limiting enzyme of histidine catabolism, we next examined whether circulating concentrations of histidine and its downstream metabolite glutamate differed across groups. Compared with healthy controls with low behavioral problems, serum levels of histidine and glutamate were significantly lower in children with asthma and high behavioral problems (Figure 3E,F). Higher protein levels of HAL and MAD1L1 showed modest correlations with lung function and BHR at age 7 (Figure 4A–C). Their corresponding metabolites, glutamate and histidine, also showed modest correlations with selected CBCL categories, including somatic complaints and anxiety/depression, assessed at ages 2, 4, and 6 (Figure 4D–F).

## 4 DISCUSSION

In this study, we demonstrated that persistently high internalizing behavior problems trajectories during the preschool period showed a dose‐dependent cumulative trend with asthma risk, with the highest risk observed among children with consistently elevated symptoms from early life, compared with those exhibiting a later increase in symptoms. This pattern from birth cohorts suggests that internalizing problems present early in life may be particularly relevant to subsequent asthma risk and highlight early life as a potentially sensitive period. Mental health problems in preschool children significantly increase the risk of childhood asthma, with blood DNA methylation suggesting a shared mechanism. Results from the COCOA and PSKC studies consistently link elevated anxiety, depression, somatic complaints, and withdrawal during preschool years to later childhood asthma development (Figure S3). DNA‐methylation analysis revealed hypomethylation of *HAL* and *MAD1L1* in children with internalizing problems and asthma. This was paralleled by higher circulating HAL protein and lower plasma histidine concentrations– findings that are biologically consistent with increased histidase activity, although causality cannot be established from these observational data. These alterations were also observed in relation to internalizing problems. Taken together, these findings suggest that early‐life behavioral problems may precede the development of childhood asthma as neuro‐airway crosstalk or a shared pathway between mental health and asthma (Figure S4).

Although observational clinical studies have shown an association of allergic disease with mental health, the causal relationship remains unclear due to study limitations of cross‐sectional design, confounding factors, or potential reverse causality.^9^ The National Asthma Campaign Manchester Asthma and Allergy Study (NACMAAS), a prospective cohort study of children, demonstrated that early‐life behavioral problems preceded wheezing onset.^10^ However, its cross‐sectional analysis only has two points for wheeze evaluation in preschool age, wherein preschool wheezing is not consistently aligned with asthma. The German birth cohort studies, GINIplus and LISA, demonstrated that psychopathological symptoms at age 11 increased the risk of developing asthma by age 15.^12^ However, this association may have been influenced by pubertal stage, and it cannot be ruled out that some children already had underlying allergic comorbidities or latent asthma susceptibility before age 11. Since previous studies did not evaluate early‐life stress exposures using longitudinal trajectory modeling of behavioral symptoms in relation to later asthma development, our study aimed to overcome these limitations by applying a trajectory‐based approach to elucidate the causal relationship between early CBCL patterns and school‐age asthma. No research has provided an explanatory mechanism for this relationship between mental health problems and childhood asthma.

Mechanisms related to allergic diseases and mental health have mainly been reported from *in vitro* studies, wherein chronic inflammation was the main mechanism. For example, stress activates the mast cell, a unique tissue immune cell that releases vasoactive and proinflammatory mediators such as prostaglandins, chemokines, cytokines, and vascular endothelial growth factor.^23^ Mediators that disrupt the blood–brain barrier (BBB) induce “allergy of the brain”, which contributes to the pathogenesis of neurodevelopmental diseases such as autism spectrum disorder.^24^ Furthermore, as the mast cell is a well‐known mediator of allergic reactions, a shared pathway exists for allergy and neurodevelopmental problems.^25^ The early‐life environment may affect DNA‐methylation profiles of common gene expressions that mediate mechanisms of allergy and neurodevelopmental disorders.^26^ In a mouse model, among offspring with allergic asthma, those with low social interaction and ASD‐like behaviors revealed differentially methylated regions enriched for immune‐signaling pathways in the microglia.^27^

Our results confirm that persistent internalizing behavioral problems and asthma comprise *HAL* hypomethylation as a shared mechanism. Histidine, an essential amino acid, is a precursor of histamine synthesis.^28^ Brain histamine, produced by histidine decarboxylase in histaminergic neurons, influences various functions, including allergic reactions, gastric acid secretion, and neurotransmission. *HAL* encodes histidase, an enzyme that breaks down histidine into urocanic acid. Neuronal histamine plays a role in physiological functions such as anxiety, stress response, appetite regulation, and the sleep–wake cycle.^29^ We demonstrated that *HAL* hypomethylation upregulates histidase function, which decreases the histamine level. *MAD1L1* is associated with psychiatric disorders such as schizophrenia, bipolar disorder, and depression^30^, ^31^ and with the inflammatory stimulation of macrophages, T‐cell activation, and pulmonary fibrosis.^32^ Our results, together with the results of these previous studies, imply the possibility of a phenotype of stress‐induced asthma among the many pathophysiological mechanisms of asthma.

One of the strengths of our study is the prospective general‐population‐based birth‐cohort design and the significant replicative clinical findings in two independent birth cohorts. In particular, the trajectory analysis strengthened our hypothesis by demonstrating that the probability of asthma increased in groups with consistently high mental health problems. Moreover, our study objectively evaluated the CBCL, a globally standardized assessment tool for children. Although the CBCL relies on maternal responses at the preschool age, we addressed this potential confounder by adjusting for maternal depression. A diagnosis of asthma in school‐aged children relies on doctor records, which enhance the reliability of caregiver surveys. Additionally, adjusting for wheezing episodes before 2 years of age strengthened the association between early‐life mental health problems and childhood asthma because wheezing during infancy is not always associated with asthma. Finally, our study demonstrates the changes in DNA methylation, gene expression, and metabolite associated with behavior problems such as anxiety, depression, and somatization.

The mechanistic sub‐study was based on a small, purposively selected sample (*n* = 39) and is exploratory and hypothesis‐generating; the findings require validation in larger, independently sampled cohorts and in functional studies. To partially address this, we performed multi‐point verification by measuring DNA methylation, proteins, and metabolites. Reflecting this small sample, no formal multiple‐testing correction was applied to the exploratory molecular screen. In addition, direct functional studies are required to elucidate the causal roles of the identified target genes in asthma pathogenesis. We did not formally adjust for medication use; in COCOA, however, medications that could affect lung function were withheld for 4 weeks before bronchial provocation testing at 7 years, in line with the standardized cohort protocol.^14^ Psychotropic medication use was not investigated; given its low frequency and the young age of the children, residual confounding from this source is likely limited, although it remains a potential limitation.

In conclusion, early‐life internalizing behavioral problem trajectories precede the development of childhood asthma at school age after adjusting for early‐life wheeze from two independent birth cohorts. This preceding relationship may be mediated by DNA hypomethylation, especially in *HAL* and *MAD1L1*. As the global trend of increasing psychological problems may constitute risk factors for asthma, the assessment of behavioral and mental health problems in young preschool children and the development of early biomarkers or identification of susceptible children will be needed to prevent childhood asthma. Additionally, continuous early life evaluation and support for the mental health of children could contribute to reducing asthma throughout the school age.

## AUTHOR CONTRIBUTIONS

**Kyungmo Hong:** Investigation. **So‐Yeon Lee:** Conceptualization; supervision. **Jisun Yoon:** Writing – original draft; conceptualization; investigation. **Eom Ji Choi:** Investigation; validation. **Jiseon Kim:** Methodology; software; data curation. **Hea Young Oh:** Methodology; software; data curation; formal analysis. **Sungsu Jung:** Investigation; validation. **Seung‐Hwa Lee:** Conceptualization; methodology; investigation; data curation. **Youn Ho Shin:** Investigation; resources. **Da Kyeong Lee:** Methodology; visualization. **Jihyun Kim:** Conceptualization; validation. **Kyung Won Kim:** Investigation; resources; writing – review and editing. **Kyung‐Sook Lee:** Conceptualization; investigation; methodology. **Ji Soo Park:** Resources; methodology. **Kangmo Ahn:** Writing – review and editing; supervision. **Yee‐Jin Shin:** Conceptualization; methodology; investigation. **Soo‐Jong Hong:** Conceptualization; funding acquisition; project administration; writing – review and editing. **Dong In Suh:** Conceptualization; supervision; writing – review and editing.

## CONFLICT OF INTEREST STATEMENT

Jisun Yoon, Seung‐Hwa Lee, Jiseon Kim, Kyungmo Hong, So‐Yeon Lee, Hea Young Oh, Sungsu Jung, Eun Young Paek, Da Kyeong Lee, Eom ji Choi, Kun‐Baek Song, Dong In Suh, Ji Soo Park, Youn Ho Shin, Kyung Won Kim, Kango Ahn, Jihyun Kim, Yee‐Jin Shin, Kyung‐Sook Lee, and Soo‐Jong Hong have no conflicts of interest to declare.

## Supporting information


Appendix S1.

## Data Availability

The data that support the findings of this study are available from the corresponding author upon reasonable request.
