# Supplementary material for: Early‐life internalizing behavioral trajectories and childhood‐onset asthma: An epigenetic link
Source: Pediatr Allergy Immunol. 2026 Aug 16;37(8):e70436. doi: 10.1111/pai.70436 (PMC13478705; doi:10.1111/pai.70436)
Supplement: Supplementary file 1 [file PAI-37-e70436-s001.docx]

**SUPPLEMENTARY MATERIAL**

**Early-Life Internalizing Behavior Trajectories and Childhood Asthma from two birth cohorts: behavioral–epigenetic pathway**

**Table of contents**

**Supplementary Table 1**

**Supplementary Table 2**

**Supplementary Table 3**

**Supplementary Table 4**

**Supplementary Table 5**

**Supplementary Figure 1**

**Supplementary Figure 2**

**Supplementary Figure 3**

**Supplementary Figure 4**

**Supplementary Methods**

**Supplementary table 1. Demographic characteristics of participants in the COCOA and PSKC studies**

| **Characteristics** | **COCOA** | **PSKC** |
| --- | --- | --- |
| **Demographic characteristics** | **N (%) or mean ± SD** | **N (%) or mean ± SD** |
| No. of participants | N=1,041 | N=1,647 |
| Sex (Male) | 535 (51·4%) | 849 (51·5%) |
| Gestational age (weeks) | 39·1±1·3 | 39·3±1·0 |
| Birth weight (kg) | 3·2±4·2 | 3·3±0·4 |
| Maternal age (years) | 32·8±3·5 | 31·3±3·7 |
| Maternal BMI (kg/m^2^) | 20·7±2·5 | 26·3±3·1 |
| Parental history of allergic disease | 505 (48·5%) | 583 (35·4%) |
| Maternal education level |  |  |
| ≤High-school | 72 (6·9%) | 501 (30·5%) |
| >High-school to ≤College | 746 (71·7%) | 1060 (64·5%) |
| >Postgraduate | 223 (21·4%) | 83 (5·0%) |
| Breastfeeding after birth (≥6 months) | 132 (12·7%) | 707 (42·9%) |
| Maternal depression |  |  |
| CES-D of children at 2, 4, and 6 years of age | 6·6±5·3/ 6·2±5·1/ 5·9±4·8 |  |
| Kessler (prenatal) |  | 11·4±3·9 |
| Any wheezing episodes (6 to 24 months) | 233 (15·1%) | 179 (12·1%) |

BMI, Body mass index; COCOA, Cohort for Childhood Origin of Asthma and allergic diseases; PSKC, Panel Study on Korean Children; CES-D, Center for Epidemiologic Studies-Depression Scale.

**Supplementary Table 2. Clinical characteristics of participants selected for DNA-methylation analysis**

| **Clinical characteristics** | **CBCL (low) & Asthma (-)** | **CBCL (low) & Asthma (+)** | **CBCL (high) & Asthma (-)** | **CBCL (high) & Asthma (+)** | ***p*-value** |
| --- | --- | --- | --- | --- | --- |
| **Number of participants** | **10** | **10** | **10** | **9** |  |
| Child’s sex (male:female) | 6:4 | 5:5 | 7:3 | 6:3 | 0·890 |
| Gestational age at birth (weeks) | 39·55±1 | 39·26±1·12 | 38·5±1·59 | 39·6±1·18 | 0·200 |
| Maternal age (years) | 31·7±3·77 | 34·3±3·13 | 32·1±2·28 | 33·56±4·19 | 0·296 |
| Birth weight (g) | 3167±326·59 | 3335±347·94 | 3171±236·38 | 3299·44±431·34 | 0·592 |
| Maternal CES-D | 4·67±4·95 | 6·86±2·85 | 5·25±3·06 | 7·75±3·65 | 0·344 |
| CBCL score at 6 years of age |  |  |  |  |  |
| Internalizing behavior | 41·9±5·7 | 49·9±6·61 | 65·2±8·95 | 67·11±9·2 | <0·001 |
| Anxious/depressed | 50·4±0·84 | 52·7±4·24 | 61·3±6·72 | 63·56±10·54 | <0·001 |
| Withdrawn/depressed | 50·9±2·85 | 52·9±4·07 | 58·7±6·57 | 60·44±6·48 | <0·001 |
| Somatic complaints | 50·8±1·75 | 54·4±4·33 | 67±4·78 | 67·22±1·92 | <0·001 |

CES-D, Center for Epidemiologic Studies-Depression Scale; CBCL, Child Behavior Checklist.

**Supplementary Table 3.** T scores of internalizing problem variables in Child Behavior Checklist (CBCL) at two, four, and six years of age between asthma and non-asthma groups (in the COCOA study)

| CBCL 1.5-5  CBCL 6-18 |  | Two years  (T-score, m±SD) | | | Four years  (T-score, m±SD) | | | Six years  (T-score, m±SD) | | |
| --- | --- | --- | --- | --- | --- | --- | --- | --- | --- | --- |
| Internalizing problem areas | Asthma definition | Asthma | Non-asthma | p-value | Asthma | Non-asthma | p-value | Asthma | Non-asthma | p-value |
| Internalizing | Asthma  Symptoms ever | 51.33±10.45 | 49.35±9.51 | 0.075 | 51.17±9.43 | 49.48±8.98 | 0.187 | 51.71±11.11 | 50.08±8.48 | 0.116 |
|  | Current asthma | 52.78±10.42 | 49.37±9.54 | 0.026* | 52.84±8.48 | 49.49±9.03 | 0.068 | 52.25±11.76 | 50.12±8.58 | 0.133 |
| Emotionally reactive | Asthma  Symptoms ever | 53.94±5.61 | 53.02±4.85 | 0.107 | 54.41±5.42 | 54.21±5.62 | 0.805 |  |  |  |
|  | Current asthma | 54.56±5.44 | 53.03±4.89 | 0.051 | 54.80±5.07 | 54.20±5.62 | 0.602 |  |  |  |
| Anxious/depressed | Asthma  Symptoms ever | 54.49±6.66 | 53.46±5.22 | 0.178 | 55.54±6.44 | 53.41±5.22 | 0.022* | 54.67±7.28 | 54.26±5.75 | 0.561 |
|  | Current asthma | 55.41±7.53 | 53.46±5.23 | 0.108 | 56.04±7.29 | 53.49±5.25 | 0.095 | 54.58±7.05 | 54.28±5.84 | 0.759 |
| Somatic complaints | Asthma  Symptoms ever | 55.14±5.40 | 53.42±4.88 | 0.003* | 53.81±6.04 | 53.32±5.12 | 0.506 | 54.64±6.17 | 52.80±4.56 | 0.001* |
|  | Current asthma | 55.32±5.52 | 53.49±4.91 | 0.02* | 54.40±6.50 | 53.32±5.14 | 0.310 | 55.55±6.66 | 52.84±4.61 | <0.001* |
| Withdrawn/depressed | Asthma  Symptoms ever | 53.47±5.50 | 53.59±5.51 | 0.856 | 53.65±5.05 | 52.80±4.73 | 0.210 | 54.51±6.37 | 53.27±4.84 | 0.096 |
|  | Current asthma | 53.83±5.57 | 53.56±5.51 | 0.763 | 54.04±5.40 | 52.82±4.73 | 0.210 | 54.58±7.55 | 53.32±4.85 | 0.304 |

COCOA; Cohort for Childhood Origin of Asthma and allergic diseases, *p-value <0.05

**Supplementary Table 4.** T scores of internalizing problem variables in Child Behavior Checklist (CBCL) at four, and seven years of age between asthma and non-asthma groups (in PSKC study)

| CBCL 1.5-5  CBCL 6-18 |  | Four years  (T-score, m±SD) | | | Seven years  (T-score, m±SD) | | |
| --- | --- | --- | --- | --- | --- | --- | --- |
| Internalizing problem areas | Asthma definition | Asthma | Non-asthma | p-value | Asthma | Non-asthma | p-value |
| Internalizing | Asthma  Symptoms ever | 50.02±9.82 | 49.84±9.58 | 0.855 | 50.8±8.96 | 49.56±8.71 | 0.092 |
|  | Current asthma | 51.72±9.42 | 49.83±9.61 | 0.200 | 52.91±10.82 | 49.56±8.64 | 0.045* |
| Emotionally reactive | Asthma  Symptoms ever | 53.40±5.08 | 53.52±5.33 | 0.811 | 53.68±5.59 | 53.73±5.44 | 0.924 |
|  | Current asthma | 53.60±4.64 | 53.53±5.34 | 0.919 | 54.71±6.76 | 53.68±5.40 | 0.208 |
| Anxious/depressed | Asthma  Symptoms ever | 53.77±5.03 | 54.00±5.66 | 0.651 | 54.41±6.04 | 52.74±4.92 | 0.005* |
|  | Current asthma | 54.84±5.65 | 53.99±5.64 | 0.336 | 55.42±7.24 | 53.06±4.95 | 0.002* |
| Somatic complaints | Asthma  Symptoms ever | 54.83±7.13 | 53.66±5.57 | 0.045 | 53.59±5.41 | 53.39±4.99 | 0.647 |
|  | Current asthma | 55.00±7.34 | 53.72±5.66 | 0.264 | 54.80±6.08 | 53.36±4.98 | 0.057 |
| Withdrawn/depressed | Asthma  Symptoms ever | 53.71±5.68 | 54.00±5.61 | 0.620 | 50.8±8.96 | 49.56±8.71 | 0.092 |
|  | Current asthma | 54.16±6.57 | 53.98±5.58 | 0.856 | 52.91±10.82 | 49.56±8.64 | 0.045* |

PSKC, Panel Study on Korean Children, *p-value <0.05

**Supplementary Table 5. Differential methylated genes associated with higher behavioral problems in asthma**

| **No.** | **Methylation** | **CpG site** | **Delta mean** | ***p*-value** | **Gene** | **Location** |
| --- | --- | --- | --- | --- | --- | --- |
| 1 | hypo | cg08823359 | −0·216 | 0·001 | *PAPSS1* | TSS200 |
| 2 | hypo | cg16490124 | −0·391 | 0·002 | *SNORA14B* | TSS1500 |
| 3 | hypo | cg17422692 | −0·260 | 0·003 | *MRPL28* | 5'UTR |
| 4 | hypo | cg15895398 | −0·235 | 0·004 | *MEGF6* | Body |
| 5 | hypo | cg08923669 | −0·224 | 0·004 | *MRPL28* | 5'UTR |
| 6 | hypo | cg18714913 | −0·222 | 0·006 | *HIVEP3* | Body |
| 7 | hypo | cg08296601 | −0·225 | 0·008 | *MRPL28* | 5'UTR |
| 8 | hypo | cg23392381 | −0·360 | 0·011 | *CTNNA2* | Body |
| 9 | hypo | cg00101154 | −0·265 | 0·011 | *MRPL28* | Body |
| 10 | hypo | cg16178271 | −0·315 | 0·017 | *MAD1L1* | Body |
| 11 | hypo | cg05786381 | −0·326 | 0·022 | *FAM53A* | 5'UTR |
| 12 | hypo | cg04534902 | −0·342 | 0·025 | *HAL* | 5'UTR |
| 13 | hypo | cg14711243 | −0·244 | 0·028 | *ACAD9* | TSS1500 |
| 14 | hypo | cg05107650 | −0·282 | 0·034 | *ZBTB38* | 5'UTR |
| 15 | hypo | cg01655658 | −0·214 | 0·045 | *HLA-L* | Body |
| 16 | hypo | cg17718984 | −0·300 | 0·045 | *MAD1L1* | ExonBnd |
| 17 | hypo | cg15059639 | −0·249 | 0·047 | *MYO3B* | Body |
| 18 | hypo | cg17187785 | −0·309 | 0·060 | *NTM* | Body |
| 19 | hypo | cg23855802 | −0·238 | 0·072 | *ADAMTS17* | Body |
| 20 | hypo | cg14361804 | −0·249 | 0·087 | *TNFSF8* | TSS1500 |
| 21 | hypo | cg18673341 | −0·258 | 0·109 | *MGC87042* | Body |
| 22 | hypo | cg26954695 | −0·301 | 0·174 | *MZF1* | TSS1500 |
| 23 | hypo | cg02866897 | −0·269 | 0·180 | *PPP1R16B* | Body |
| 24 | hypo | cg18172516 | −0·244 | 0·224 | *RBMS1* | Body |
| 25 | hypo | cg15979214 | −0·253 | 0·263 | *TRIM10* | Body |
| 26 | hyper | cg15025170 | 0·301 | 0·000 | *TBX5* | 5'UTR |
| 27 | hyper | cg12710220 | 0·245 | 0·001 | *PRKAG2* | Body |
| 28 | hyper | cg07460804 | 0·247 | 0·004 | *MKL2* | TSS1500 |
| 29 | hyper | cg00418190 | 0·278 | 0·005 | *ELK3* | Body |
| 30 | hyper | cg16458822 | 0·340 | 0·005 | *ENOX1* | 5'UTR |
| 31 | hyper | cg11265839 | 0·213 | 0·008 | *ELK3* | Body |
| 32 | hyper | cg01264919 | 0·345 | 0·008 | *MUC5B* | Body |
| 33 | hyper | cg06638795 | 0·196 | 0·009 | *KCNG3* | Body |
| 34 | hyper | cg14575022 | 0·262 | 0·009 | *ELK3* | Body |
| 35 | hyper | cg01347786 | 0·345 | 0·010 | *RBMS3* | Body |
| 36 | hyper | cg18756775 | 0·289 | 0·012 | *ELK3* | Body |
| 37 | hyper | cg09331844 | 0·202 | 0·014 | *GYS2* | Body |
| 38 | hyper | cg01927686 | 0·300 | 0·019 | *ELK3* | Body |
| 39 | hyper | cg08208480 | 0·251 | 0·021 | *BRSK2* | Body |
| 40 | hyper | cg18075755 | 0·417 | 0·023 | *C1QTNF7* | 5'UTR |
| 41 | hyper | cg19270931 | 0·324 | 0·032 | *BREA2* | TSS1500 |
| 42 | hyper | cg17759595 | 0·175 | 0·033 | *DPYSL2* | Body |
| 43 | hyper | cg15465743 | 0·255 | 0·037 | *BRSK2* | Body |
| 44 | hyper | cg17658113 | 0·218 | 0·039 | *BRSK2* | Body |
| 45 | hyper | cg16254485 | 0·219 | 0·043 | *C5orf49* | TSS1500 |
| 46 | hyper | cg23803868 | 0·295 | 0·045 | *SPPL2B* | Body |
| 47 | hyper | cg06378142 | 0·292 | 0·056 | *PRR12* | Body |
| 48 | hyper | cg17682313 | 0·394 | 0·066 | *FBXW7* | 5'UTR |
| 49 | hyper | cg16020483 | 0·210 | 0·135 | *CASZ1* | 5'UTR |
| 50 | hyper | cg11124135 | 0·257 | 0·181 | *LSM1* | Body |
| 51 | hyper | cg18861767 | 0·211 | 0·246 | *C10orf58* | Body |

**Supplementary Figure 1. Flowchart depicting the study procedures and conduct of the study.**

**Supplementary Figure 2. Functional annotation enrichment analysis of disease terms associated with internalizing behavioral problems and asthma-relevant methylated genes in children.**

**Supplementary Figure 3. Schematic summary diagrams of internalizing behavior problems and childhood asthma from COCOA and PSKC birth cohort study. Created with BioRender.com**

**Supplementary Figure 4. Potential mechanism of the shared neuro-airway pathway. Early-life internalizing behavioral problems precede childhood-onset asthma, which is mediated by DNA methylation, especially of the histidine ammonia-lyase (*HAL*) gene, wherein the HAL protein level increased and levels of its metabolites histidine and glutamate decreased. Created with BioRender.com**

***Assessment of children’s internalizing behavioral problems***

The Childhood Behavior Checklist (CBCL) is a widely used questionnaire that assesses behavioral and emotional problems in young children and adolescents. The CBCL consists of a series of statements that describe specific behaviors or emotions, including externalizing problems (i.e., aggression and hyperactivity), internalizing problems (i.e., anxiety and depression), and other problems (i.e., somatic complaints and social withdrawal).^1^ The CBCL is designed to be completed by parents or other caregivers who know the child well and, therefore, has the advantage of facilitating the evaluation of the mental health problems of preschool-aged children.^2,3^

There are two versions of the CBCL according to the age range of the participants; accordingly, the versions for ages 1·5–5 and 6–18 years were used in this study. The CBCL includes subscales for assessing symptoms of internalizing, externalizing, and sleep problems, and DSM-oriented problem scales.^1^ Studies on the reliability and validity of the CBCL have been extensively summarized in the Achenbach manual, and studies of the Korean-CBCL version have shown good validity and reliability,^4^ with T-scores of 23–100. We evaluated the internalization subcategories of emotionally reactive, anxious/depressed, somatic complaints, and withdrawn/depressed from the CBCL scale as measures of children’s behavioral problems. Internalization scores of <60 were considered non-clinical, 60–63 were borderline, and ≥64 were clinical. Subcategories of internalization scores of <65, 65–68, and ≥69 were considered non-clinical, borderline, and clinical, respectively.

***DNA-methylation experiments***

Imaging and data analysis of these chips were performed using an Illumina iScan scanner. Each methylation data point is represented by fluorescent signals from the methylated (M) and unmethylated (U) alleles. The background intensity was computed from a set of negative controls and subtracted from each analytical data point. The fluorescent signal ratio for the two alleles was then computed as β = (max(M, 0))/(|U| + |M| + 100). The β-value reflects the methylation level of each CpG site. A β-value range of 0–1 represented 0–100% methylation, respectively, for each CpG site. Array data export processing and analysis were performed using Illumina GenomeStudio v2011·1 (Methylation Module v1·9·0) and R 3·0·2. CpG sites with a *p* <0·05 and a mean methylation change >0·2 were considered to be differentially methylated.^5^

***Quantitative Measurement of glutamate and histidine metabolites***

Glutamate, histidine, 13C5-glutamine, and derivatization reagents were purchased from Sigma-Aldrich or CDN isotopes. All solvents including water were purchased from J. T. Baker.

50μL of human plasma was mixed well with 375 μL of chloroform/methanol (1/2, v/v) and internal standard solution (10 μM of 13C5-glutamine). The sample was centrifuged at 13,000 rpm for 15 min. The supernatant was collected, and aqueous phase was collected from liquid-liquid extraction after adding 100 μL of chloroform and H2O. The aqueous phase was used for chemical derivatization of amino acids using phenylisothiocyante (PITC). After the reaction, the derivatized amino acids were extracted with 5 mM ammonium acetate in methanol, and ready for LC-MS/MS analysis.

Amino acids were analyzed with LC-MS/MS equipped with 1290 HPLC (Agilent) and Triple quad 6500 (AB Sciex). 3 μL was injected into the LC-MS/MS system and ionized with a turbo spray ionization source. A reverse phase column (Zorbax Eclipse XDB-C18 100 × 2 mm) was used with 0.2 % formic acid in H2O and 0.2 % formic acid in acetonitrile as mobile phase A and B, respectively. The separation gradient was as follows: hold at 0 % B for 0.5 min, 0 to 95 % B for 5 min, 95 % B for 1 min, and 95 to 0 % B for 0.5 min, then hold at 0 % B for 2.5 min. LC flow was 500 μL/min, and column temperature was kept at 50℃. Multiple reaction monitoring (MRM) was used in positive ion mode, and the extracted ion chromatogram (EIC) corresponding to the specific transition for glutamate and histidine was used for quantitation. Calibration range was 0.12 μM–1200 μM for glutamate and 0.005 μM–500 μM for histidine with R2>0.99. Data analysis was performed by using Analyst 1.7.1 software.

***Enzyme-linked immunosorbent assay (ELISA) for measurement of HAL and MAD1L1***

The levels of histidine ammonia lyase (HAL; abx156887, Abbexa) and Mitotic Arrest Deficient 1 Like 1 (MAD1L1; abx385115, Abbexa) in the serum were evaluated using commercial enzyme-linked immunosorbent assay kits according to the manufacturer’s instructions.

**References**

1	Chang HY, Seo JH, Kim HY*, et al.* Allergic diseases in preschoolers are associated with psychological and behavioural problems. *Allergy Asthma Immunol Res* 2013; 5: 315-321.

2	Neo WS, Suzuki T, Kelleher BL. Structural validity of the Child Behavior Checklist (CBCL) for preschoolers with neurogenetic syndromes. *Res Dev Disabil* 2021; 109: 103834.

3	de la Osa N, Granero R, Trepat E*, et al.* The discriminative capacity of CBCL/1½-5-DSM5 scales to identify disruptive and internalizing disorders in preschool children. *Eur Child Adolesc Psychiatry* 2016; 25: 17-23.

4	Park E-Y, Seo H, Blair K-SC*, et al.* Rasch Analysis of the Korean-Child Behavior Checklist (K-CBCL) to Assess Emotional and Behavioral Problems in Children With Autism Spectrum Disorder. *SAGE Open* 2021; 11: 21582440211040790.

5	Kim HB, Kang MJ, Lee SY*, et al.* Prenatal maternal anxiety promotes atopic dermatitis in offspring via placental DNA methylation changes. *Asian Pac J Allergy Immunol* 2023; 41: 60-66.
